# Supplementary figures and images for: Dissecting the phase separation and oligomerization activities of the carboxysome positioning protein McdB
Source: eLife. 2023 Sep 5;12:e81362. doi: 10.7554/eLife.81362 (PMC10554743; doi:10.7554/eLife.81362)

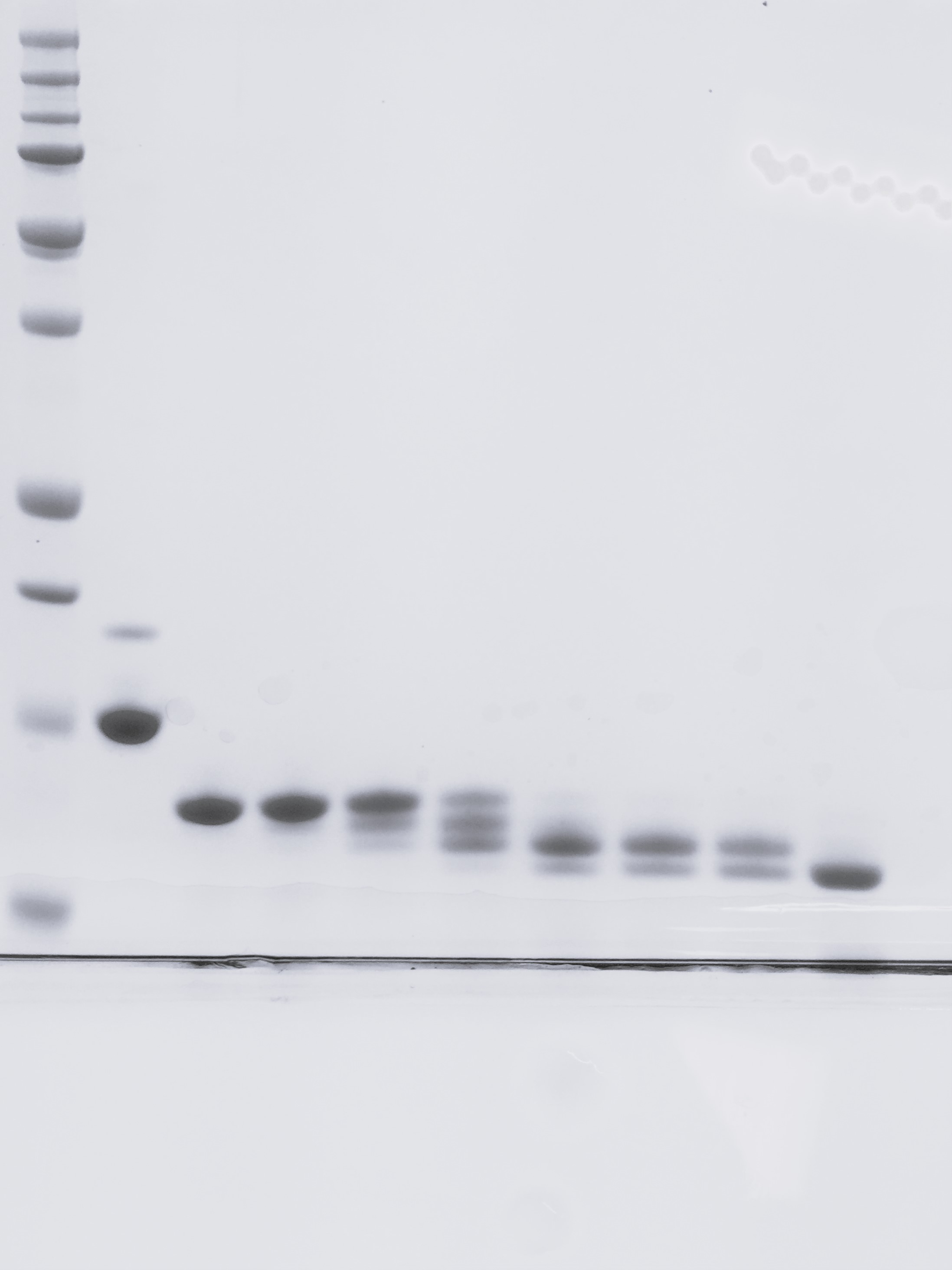

Supplement: Figure 1—source data 1. — Bands A, B, and C as well as the full-length McdB are labeled. [file elife-81362-fig1-data1.zip › Figure 1-source data 1.tif]

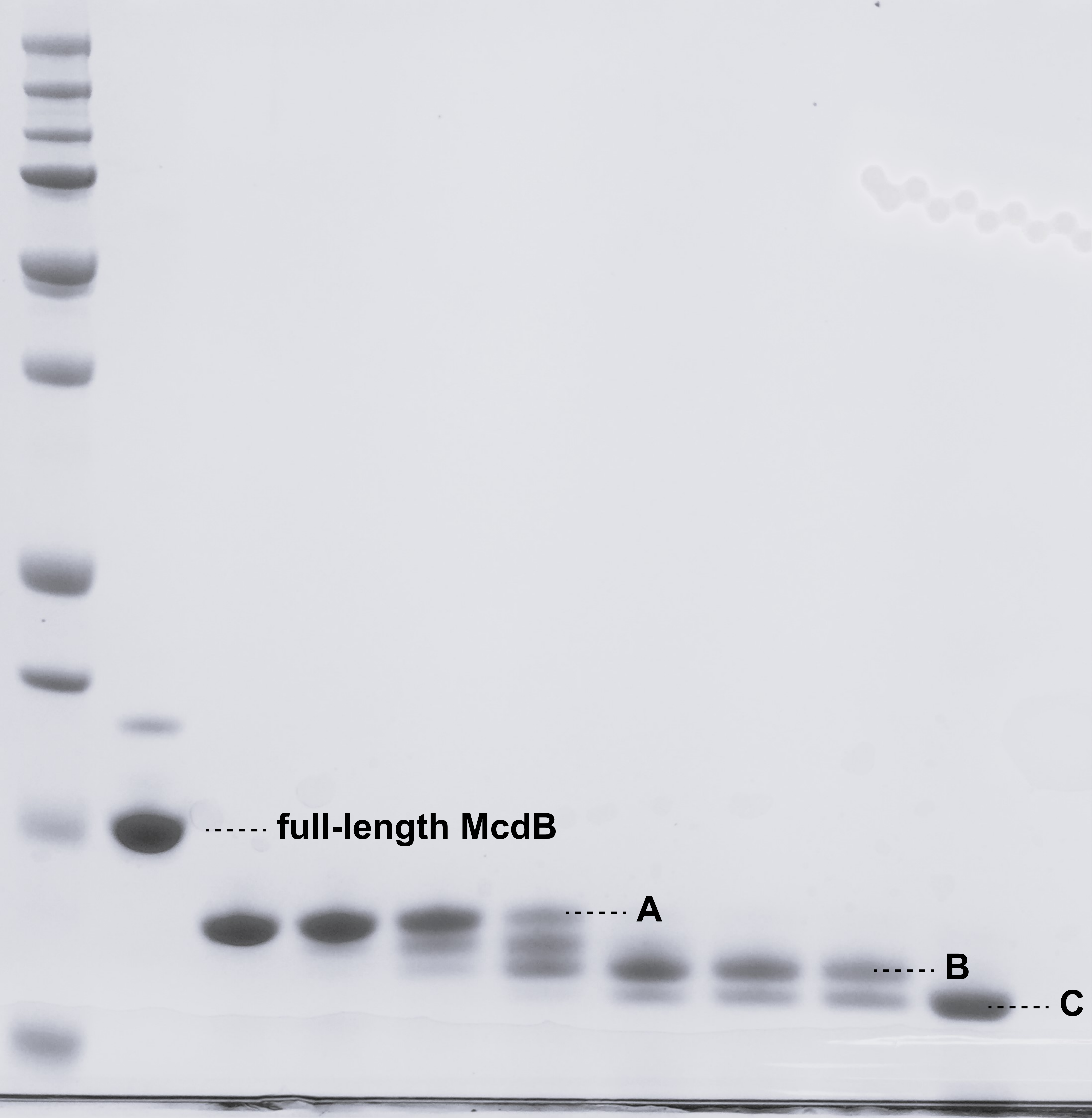

Supplement: Figure 1—source data 1. — Bands A, B, and C as well as the full-length McdB are labeled. [file elife-81362-fig1-data1.zip › Figure 1-source data 1-labeled.pdf]

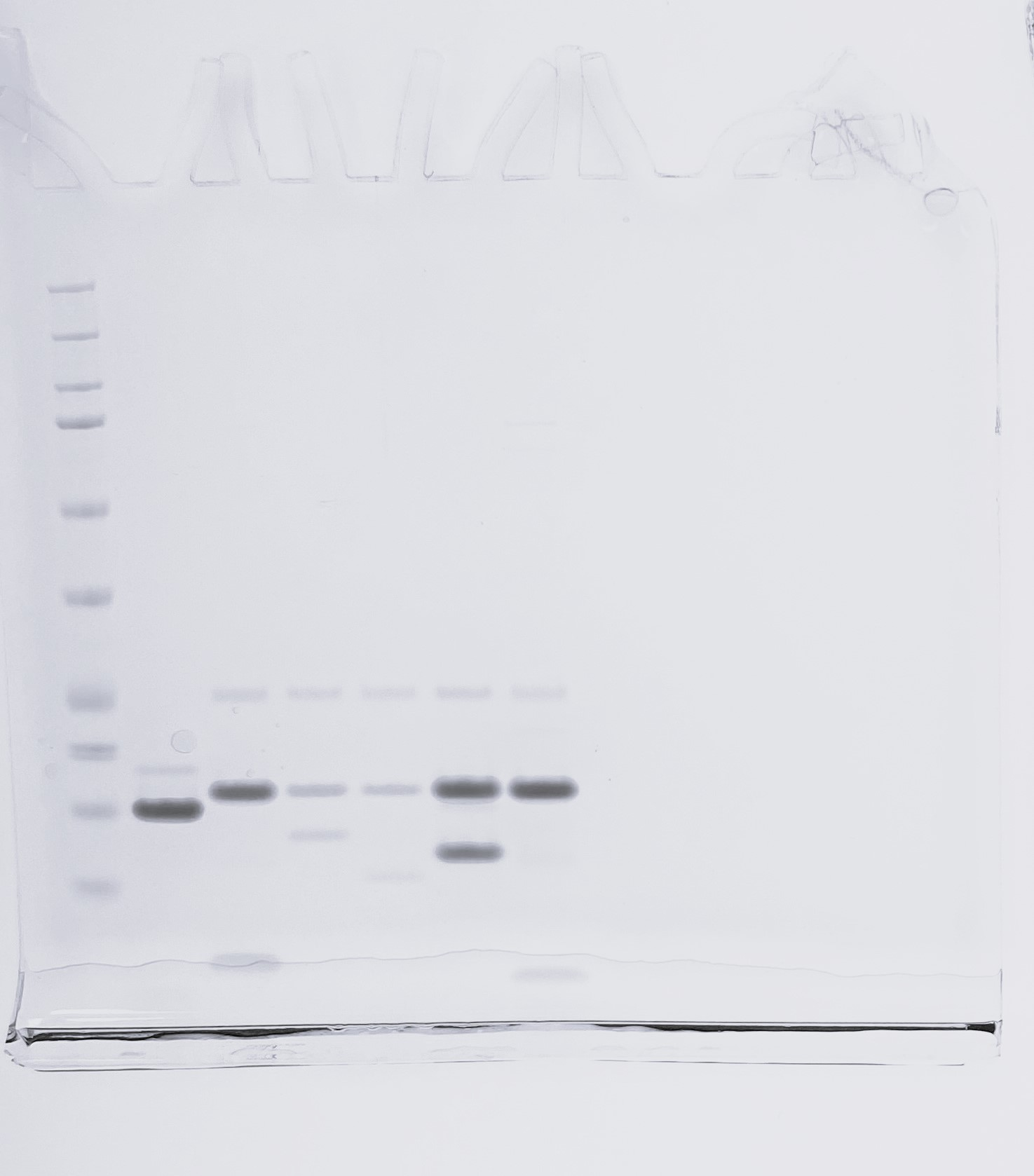

Supplement: Figure 2—figure supplement 1—source data 1. — Full-length McdB, each truncation, and the His-SUMO tag are labeled. [file elife-81362-fig2-figsupp1-data1.zip › Figure 2-figure supplement 1-source data 1.tif]

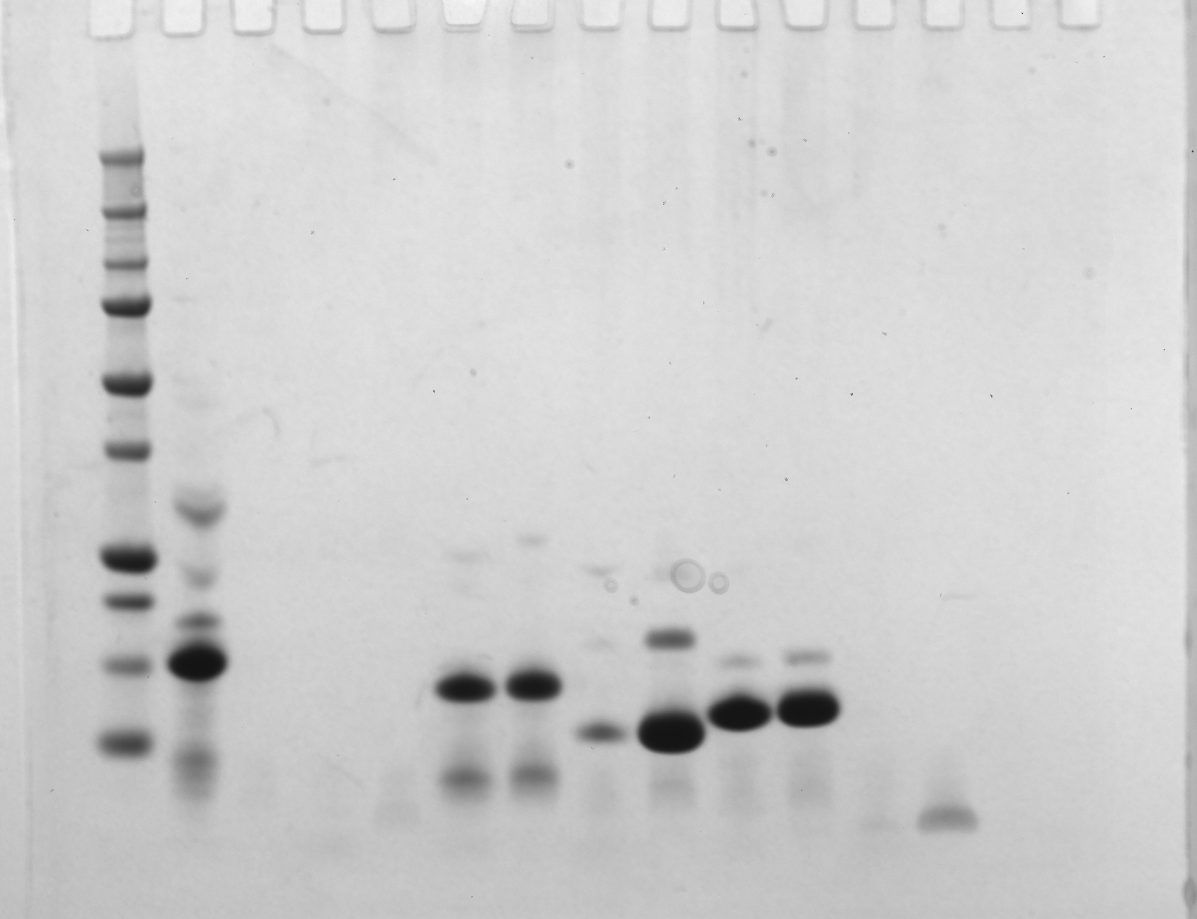

Supplement: Figure 4—source data 1. — Full-length McdB and each truncation are labeled. Bands for the pellet and supernatant fractions are labeled. [file elife-81362-fig4-data1.zip › Figure 4-source data 1.tif]

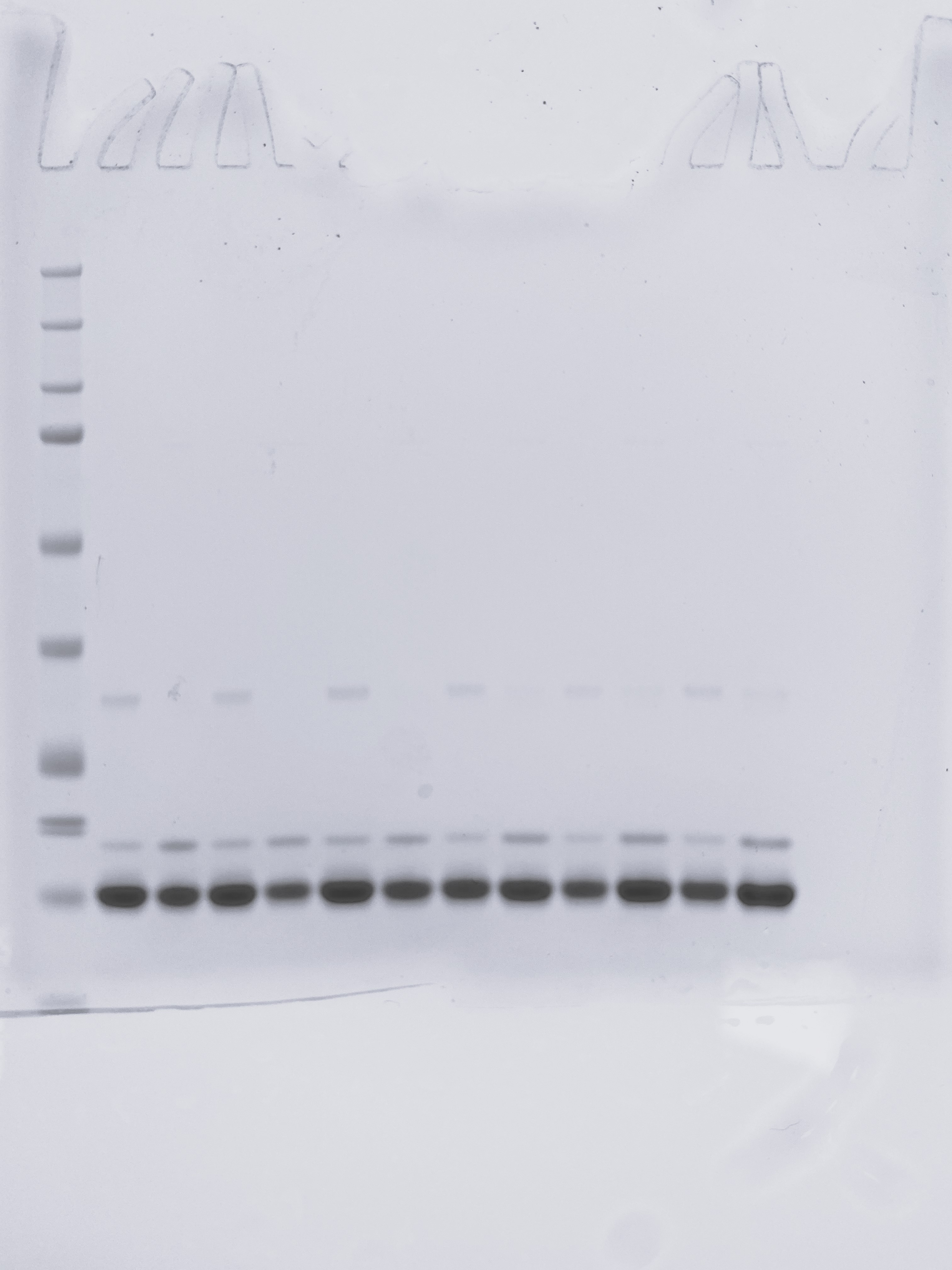

Supplement: Figure 5—source data 1. — Full-length McdB is labeled. Bands for the pellet and supernatant fractions are labeled. The corresponding KCl concentration for each condition is labeled. [file elife-81362-fig5-data1.zip › Figure 5-source data 1.tif]

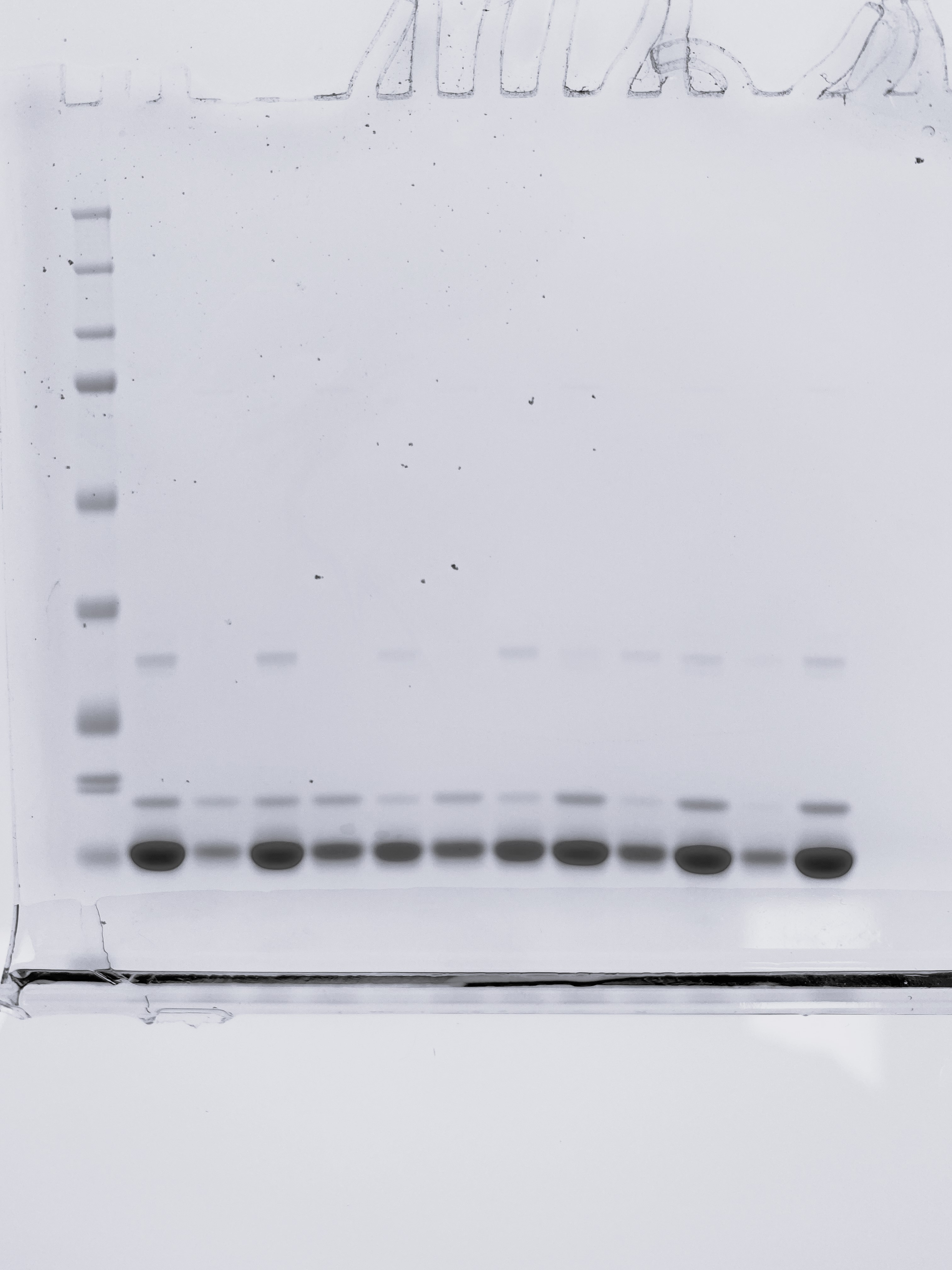

Supplement: Figure 5—source data 2. — Full-length McdB is labeled. Bands for the pellet and supernatant fractions are labeled. The corresponding pH for each condition is labeled. [file elife-81362-fig5-data2.zip › Figure 5-source data 2.tif]

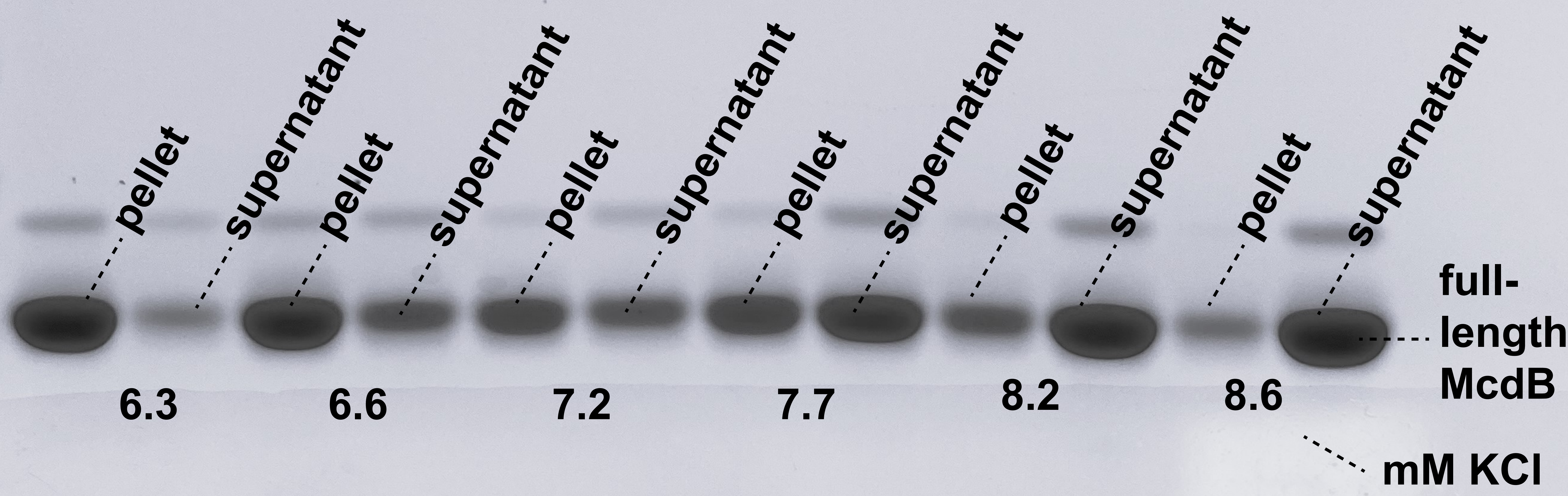

Supplement: Figure 5—source data 2. — Full-length McdB is labeled. Bands for the pellet and supernatant fractions are labeled. The corresponding pH for each condition is labeled. [file elife-81362-fig5-data2.zip › Figure 5-source data 2-labeled.pdf]

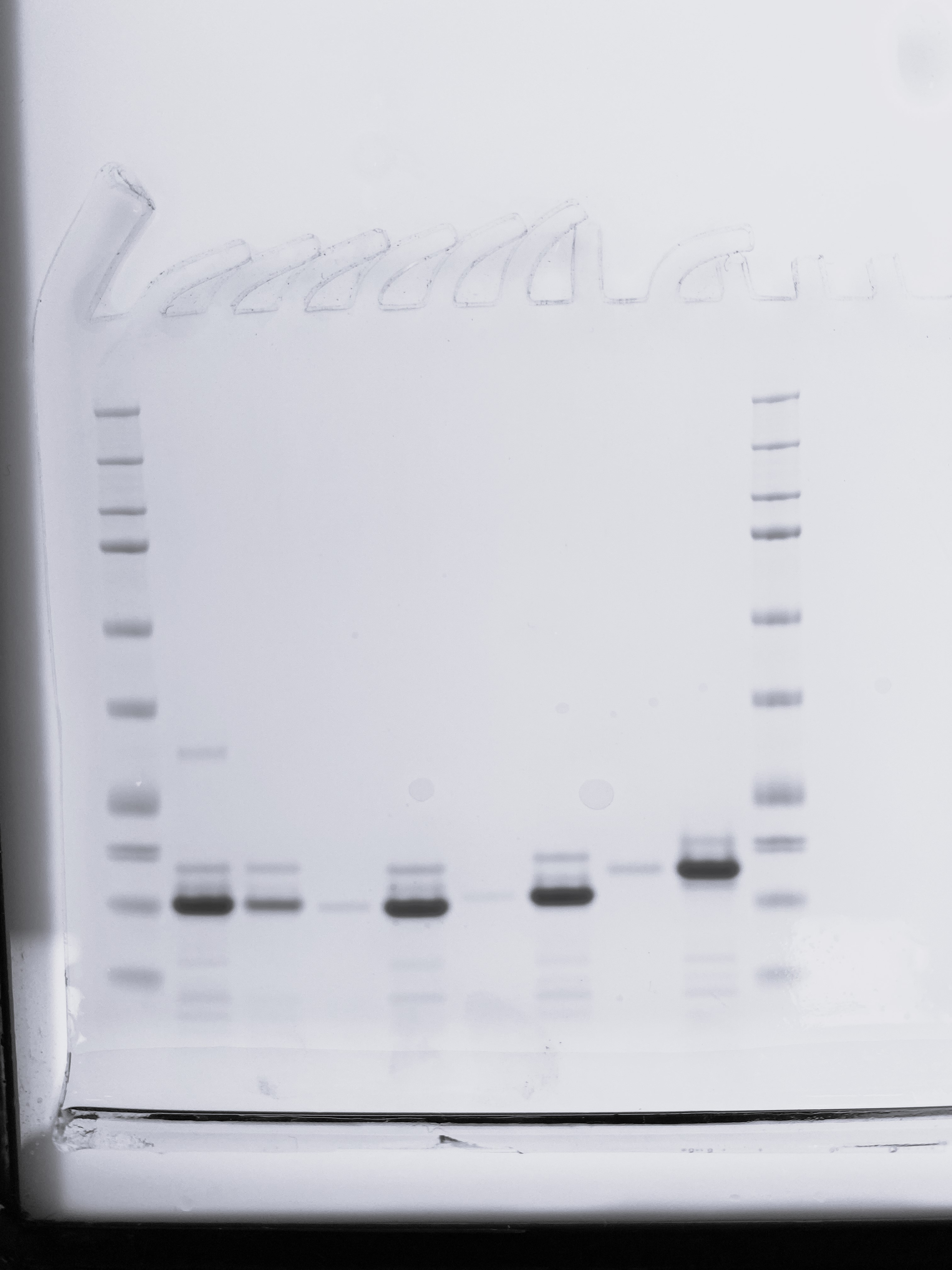

Supplement: Figure 5—source data 3. — Full-length McdB and each glutamine-substitution mutant are labeled. Bands for the pellet and supernatant fractions are labeled. [file elife-81362-fig5-data3.zip › Figure 5-source data 3.tif]

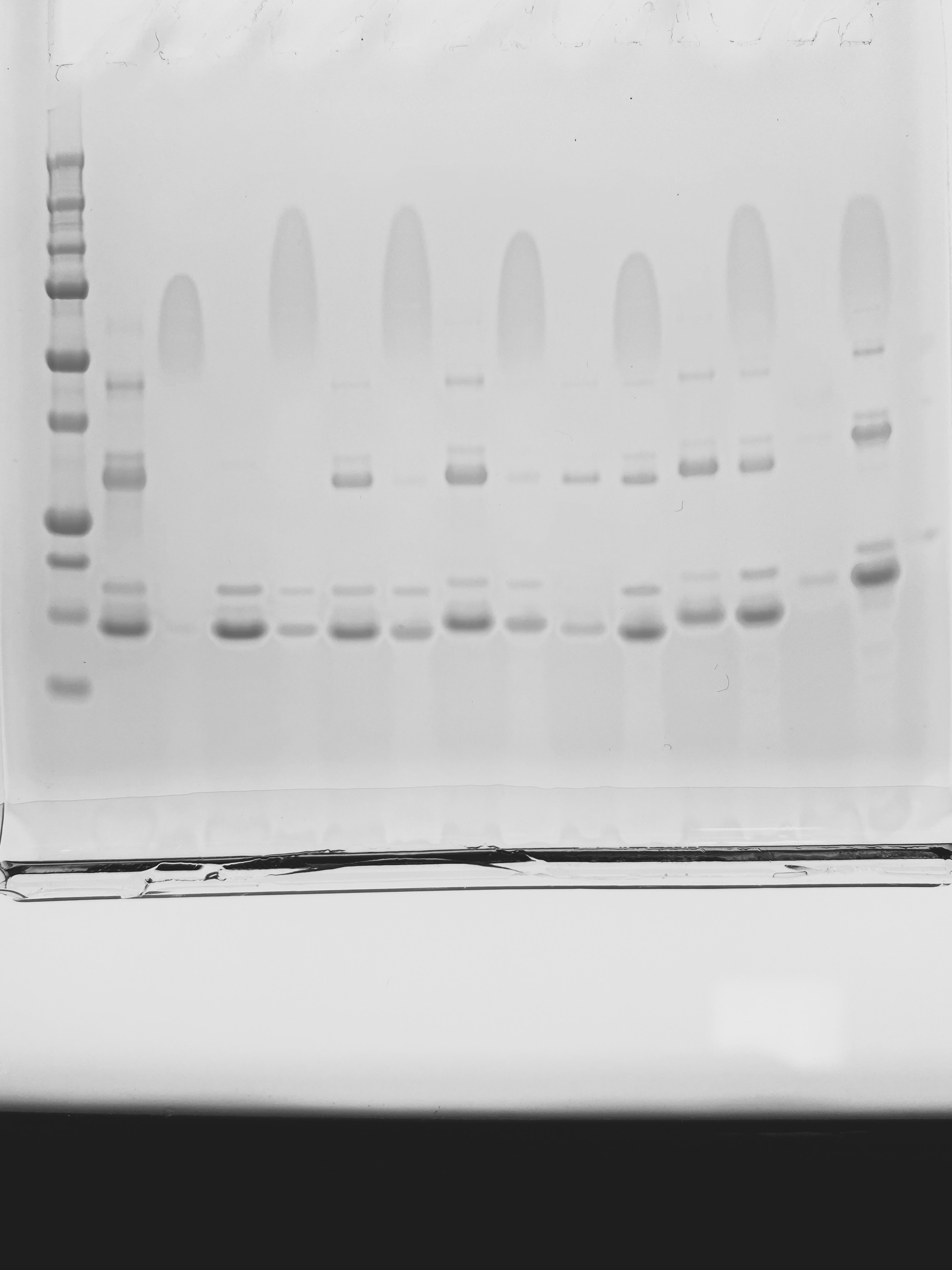

Supplement: Figure 6—source data 1. — Full-length McdB and each glutamine-substitution mutant are labeled. Bands for the pellet and supernatant fractions are labeled. [file elife-81362-fig6-data1.zip › Figure 6-source data 1.tif]

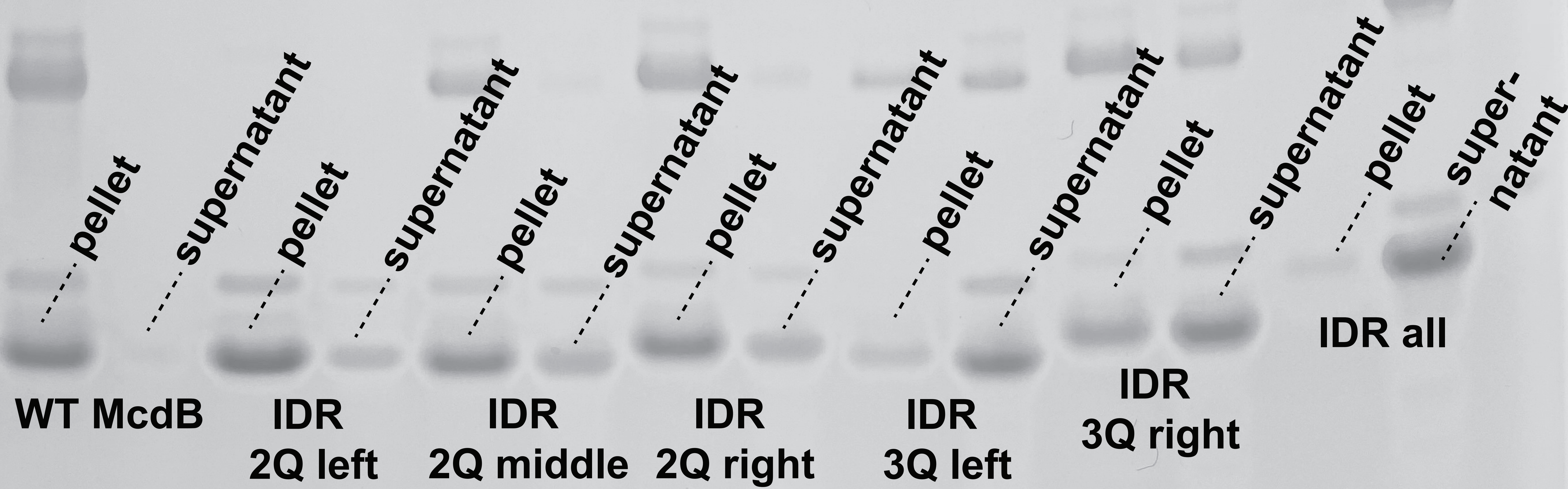

IDR all

Supplement: Figure 6—source data 1. — Full-length McdB and each glutamine-substitution mutant are labeled. Bands for the pellet and supernatant fractions are labeled. [file elife-81362-fig6-data1.zip › Figure 6-source data 1-labeled.pdf]

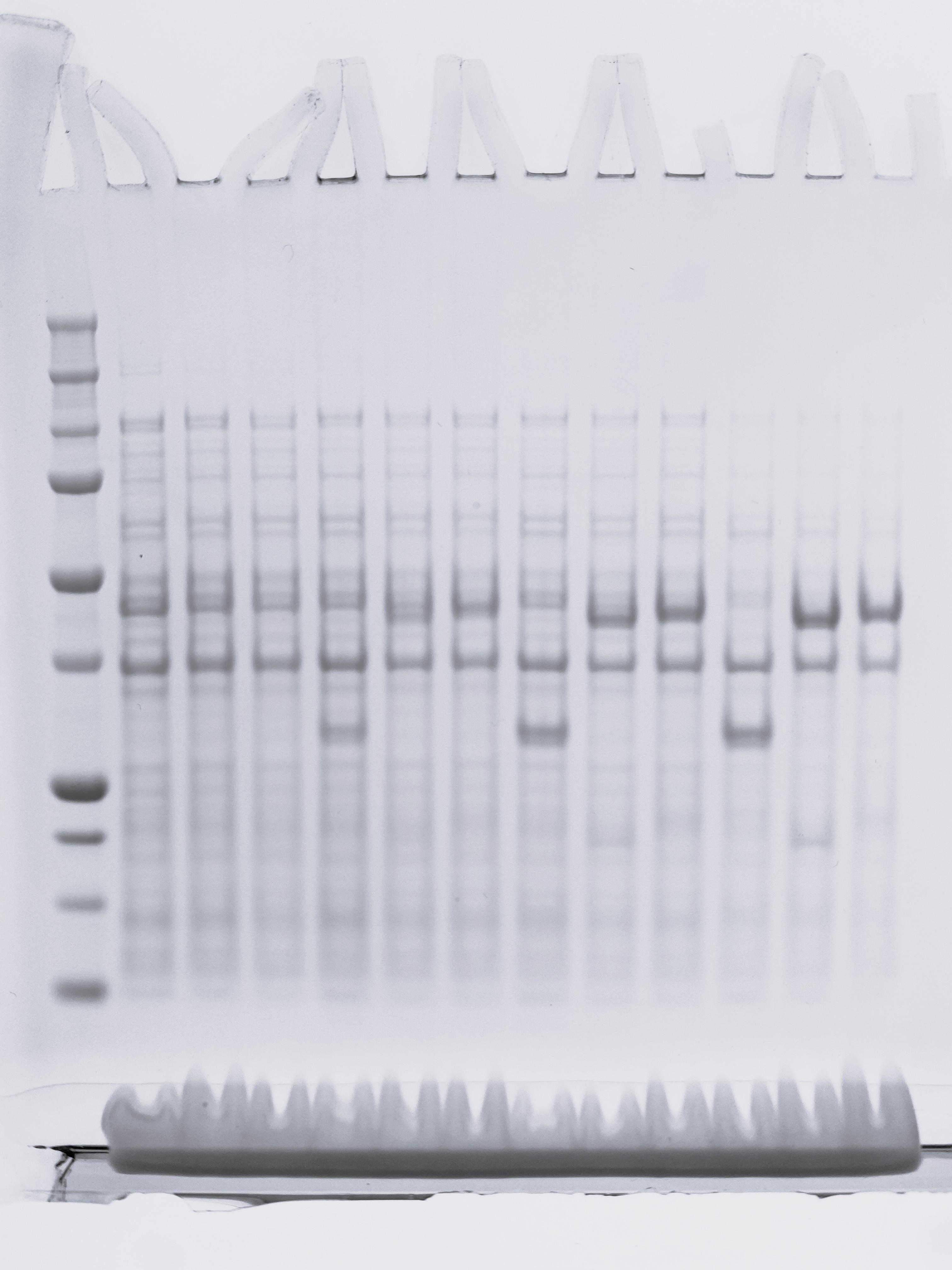

Supplement: Figure 7—source data 1. — Bands representing mCherry alone, mCh-McdB[wt], and mCh-McdB[−3] are boxed and labeled. The time after induction is indicated. [file elife-81362-fig7-data1.zip › Figure 7-source data 1.tif]

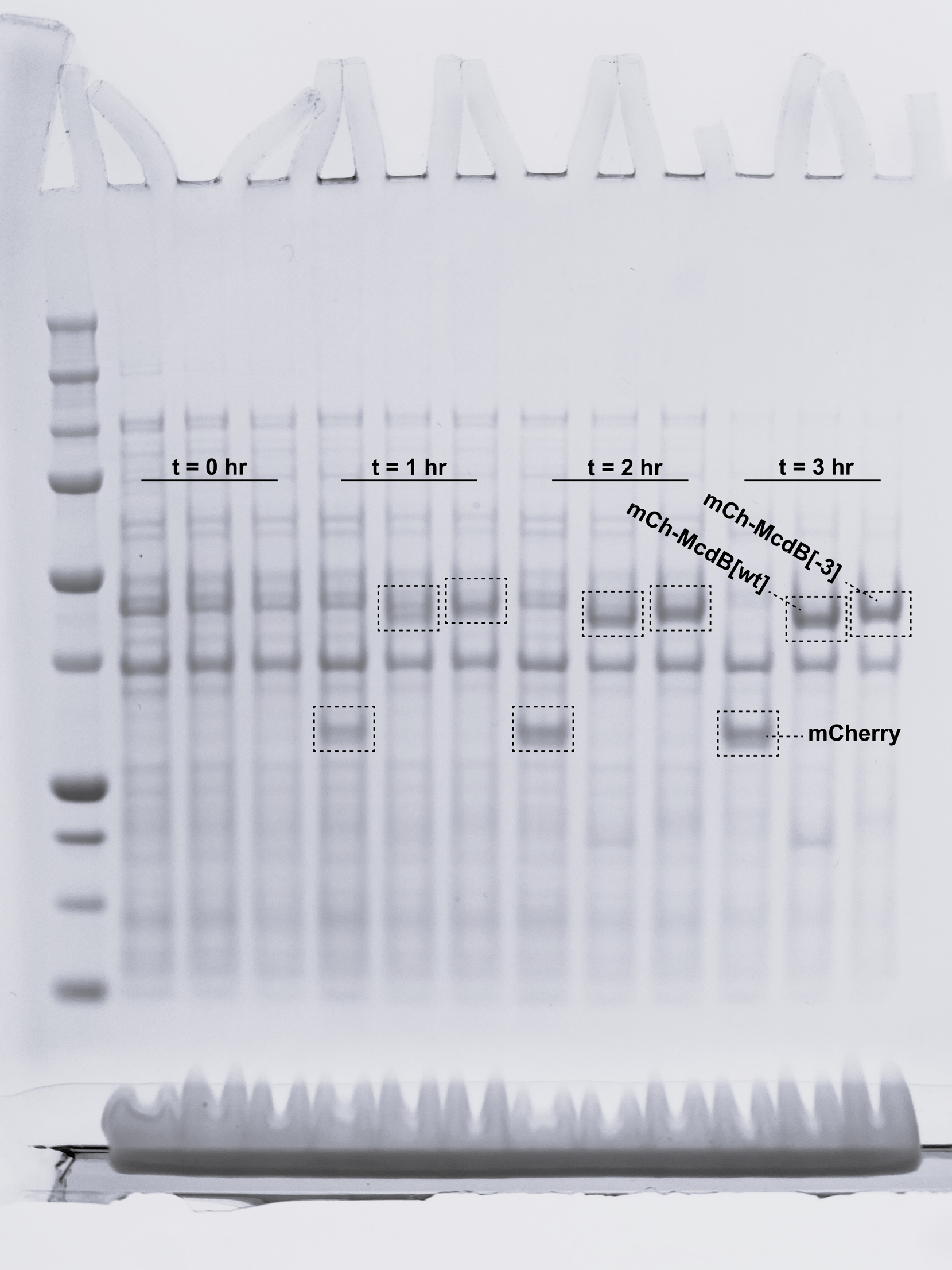

Supplement: Figure 7—source data 1. — Bands representing mCherry alone, mCh-McdB[wt], and mCh-McdB[−3] are boxed and labeled. The time after induction is indicated. [file elife-81362-fig7-data1.zip › Figure 7-source data 1-labeled.pdf]
