# Supplementary material for: Dissecting the phase separation and oligomerization activities of the carboxysome positioning protein McdB
Source: eLife. 2023 Sep 5;12:e81362. doi: 10.7554/eLife.81362 (PMC10554743; doi:10.7554/eLife.81362)
Supplement: Figure 2—figure supplement 1—source data 1. — Full-length McdB, each truncation, and the His-SUMO tag are labeled. [file elife-81362-fig2-figsupp1-data1.zip › Figure 2-figure supplement 1-source data 1-labeled.pdf]

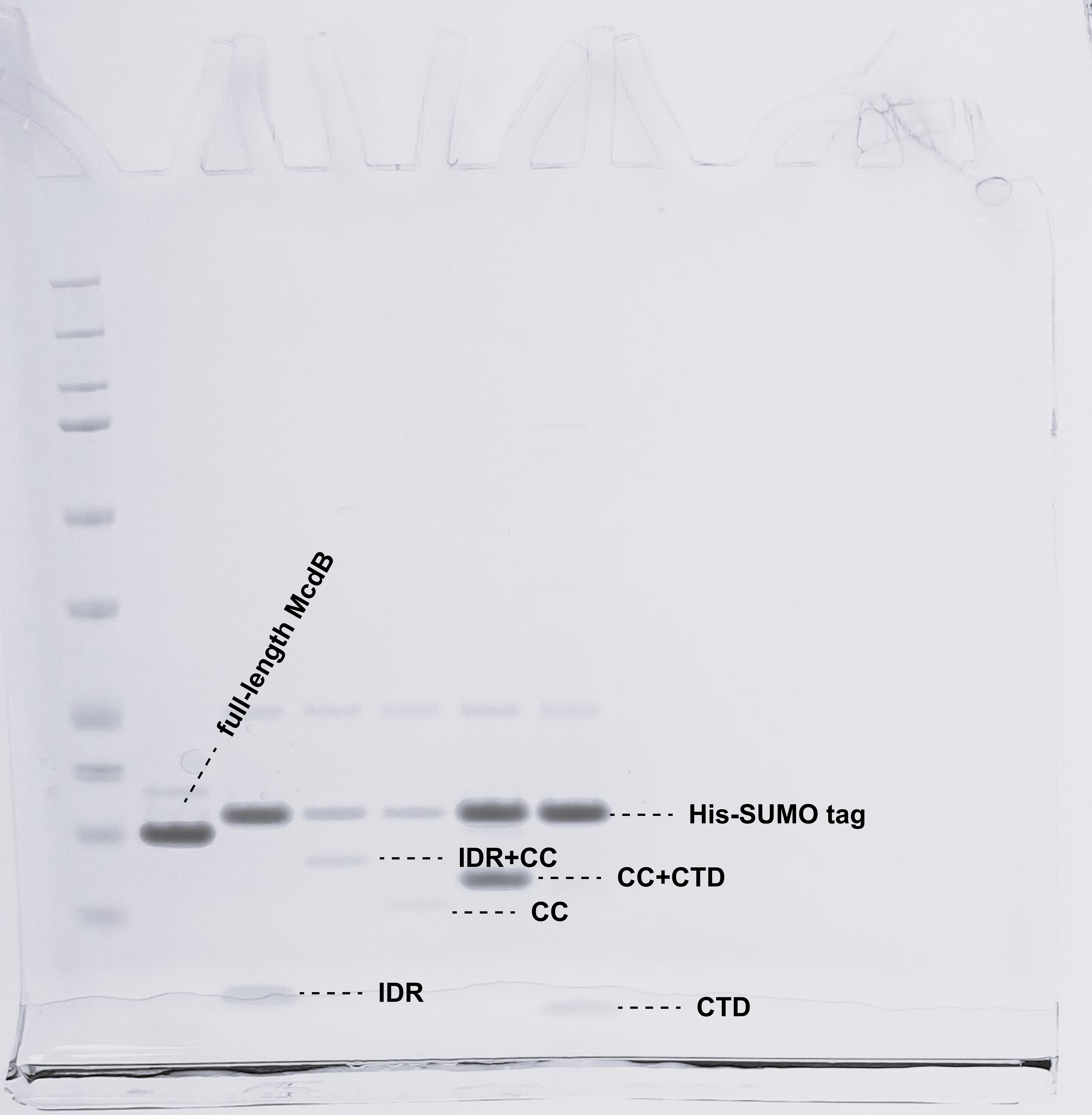
full-length McdB
His-SUMO tag
IDR+CC
CC+CTD
CC
IDR
CTD
