# Supplementary material for: Dissecting the phase separation and oligomerization activities of the carboxysome positioning protein McdB
Source: eLife. 2023 Sep 5;12:e81362. doi: 10.7554/eLife.81362 (PMC10554743; doi:10.7554/eLife.81362)
Supplement: Figure 4—source data 1. — Full-length McdB and each truncation are labeled. Bands for the pellet and supernatant fractions are labeled. [file elife-81362-fig4-data1.zip › Figure 4-source data 1-labeled.pdf]

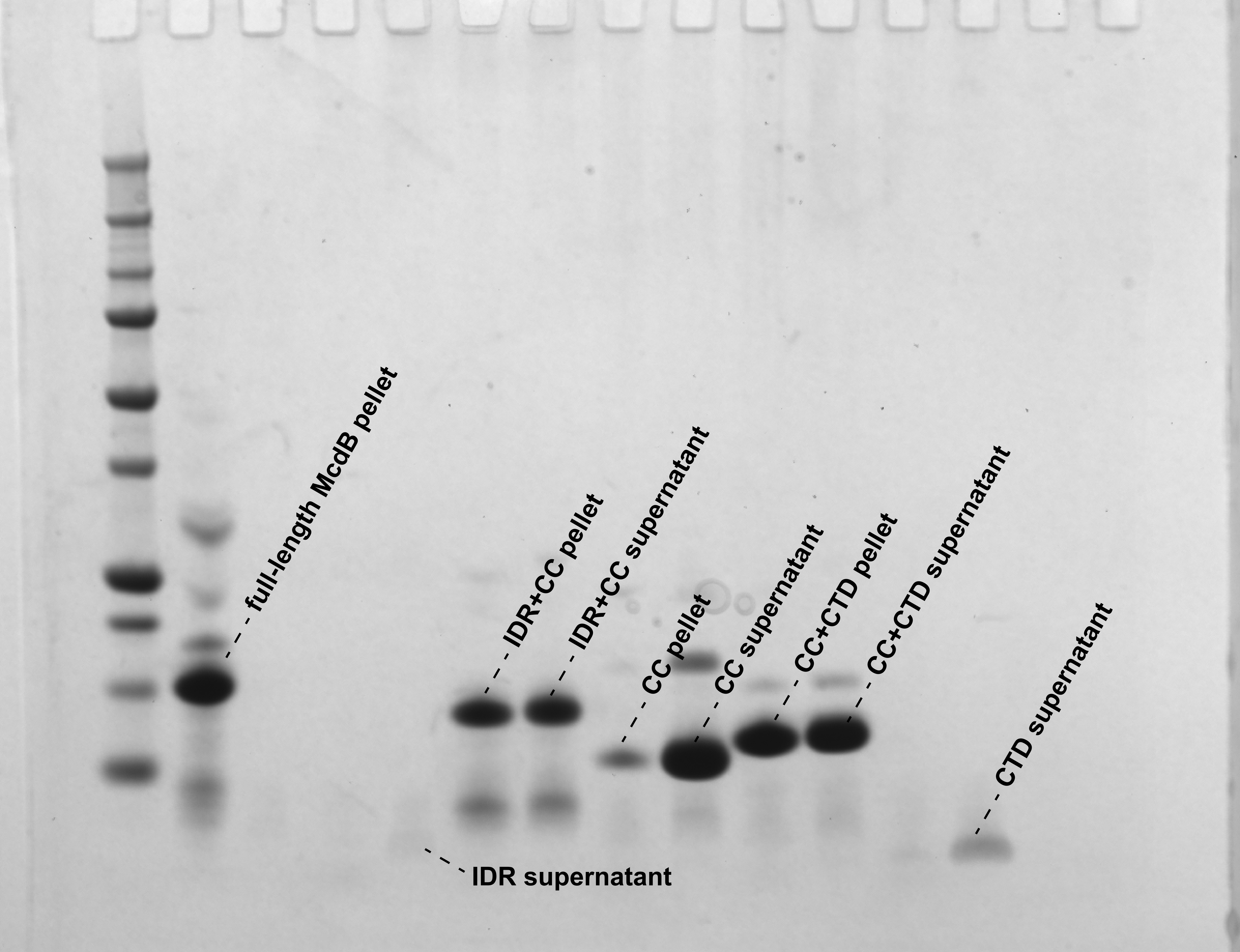

full-length McdB pellet
IDR supernatant
IDR+CC pellet
IDR+CC supernatant
CC pellet
CC supernatant
CC+CTD pellet
CC+CTD supernatant
CTD supernatant
