# Supplementary material for: Dissecting the phase separation and oligomerization activities of the carboxysome positioning protein McdB
Source: eLife. 2023 Sep 5;12:e81362. doi: 10.7554/eLife.81362 (PMC10554743; doi:10.7554/eLife.81362)
Supplement: Figure 5—source data 3. — Full-length McdB and each glutamine-substitution mutant are labeled. Bands for the pellet and supernatant fractions are labeled. [file elife-81362-fig5-data3.zip › Figure 5-source data 3-labeled.pdf]

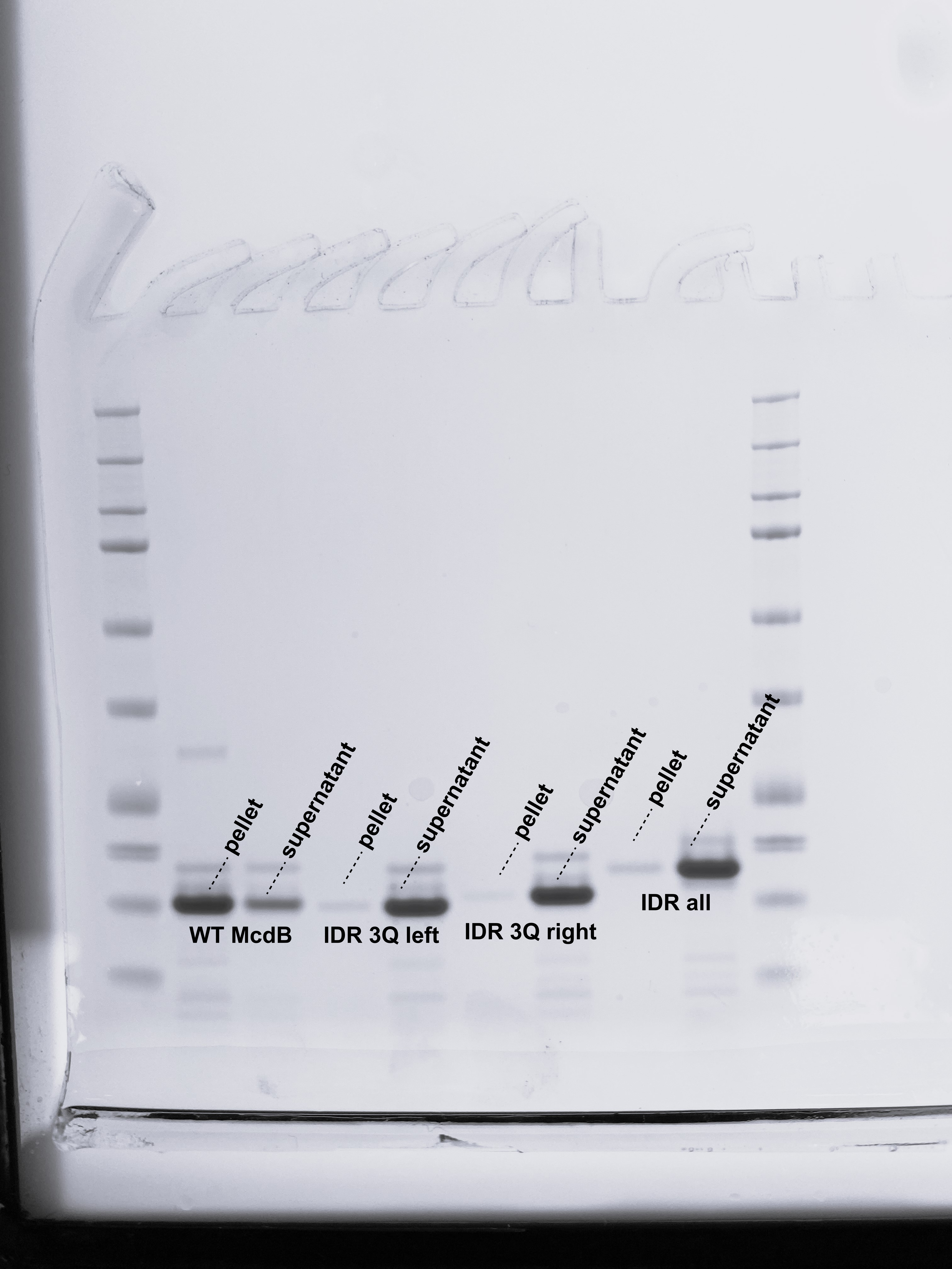
pellet
supernatant
pellet
supernatant
pellet
supernatant
pellet
supernatant
WT McdB
IDR 3Q left
IDR 3Q right
IDR all
